# The T1D Index: Implications of Initial Results, Data Limitations, and Future Development

**DOI:** 10.1007/s11892-023-01520-4

**Published:** 2023-08-23

**Authors:** Graham D. Ogle, Gabriel A Gregory, Fei Wang, Thomas IG Robinson, Jayanthi Maniam, Dianna J Magliano, Trevor John Orchard

**Affiliations:** 1 Life for a Child Program, Diabetes NSW, 26 Arundel St., Glebe, Sydney, New South Wales 2037 Australia; 2 Sydney Medical School, University of Sydney, City Rd, Camperdown, Sydney, New South Wales 2066 Australia; 3 JDRF Australia, 4/80-84 Chandos St., St Leonards, Sydney, New South Wales 2065 Australia; 4 St Vincent’s Hospital Sydney, 390 Victoria Street, Darlinghurst, Sydney, New South Wales 2010 Australia; 5 School of Medical Sciences, UNSW Sydney, Kensington, Sydney, New South Wales 2052 Australia; 6 Baker Heart and Diabetes Institute, 75 Commercial Rd, Melbourne, Victoria 3004 Australia; 7 School of Public Health and Preventive Medicine, Monash University, 553 St Kilda Rd, Melbourne, Victoria 3004 Australia; 8 Department of Epidemiology, University of Pittsburgh, School of Public Health, Pittsburgh, PA USA

**Keywords:** Type 1 diabetes, Epidemiology, Incidence, Prevalence, Diabetes-related mortality, Healthy life years

## Abstract

**Purpose of the Review:**

Current global information on incidence, prevalence, and mortality of type 1 diabetes (T1D) is limited, particularly in low- and middle-income countries. To address this gap in evidence, JDRF, Life for a Child, International Society for Pediatric and Adolescent Diabetes, and International Diabetes Federation have developed the T1D Index, which uses a Markov mathematical model, and machine learning and all available data to provide global estimates of the burden on T1D. This review assesses the methodology, limitations, current findings, and future directions of the Index.

**Recent Findings:**

Global prevalence was estimated at 8.4 million in 2021, with 1.5 million <20 years (y). T1D prevalence varied from 1.5 to 534 per 100,000, with T1D accounting for <0.1–17.8% of all diabetes in different countries. A total of 35,000 young people <25 y are estimated to have died at clinical onset of T1D from non-diagnosis. An estimated 435,000 people <25 y were receiving “minimal care.” Health-adjusted life years (HALYs) lost for individuals diagnosed with T1D at age 10 y in 2021 ranged from 14 to 55 y.

**Summary:**

These results show that interventions to reduce deaths from non-diagnosis, and improve access to at least an intermediate care level, are needed to reduce projected life years lost. The results have significant uncertainties due to incomplete data across the required inputs. Obtaining recent incidence, prevalence, and mortality data, as well as addressing data quality issues, misdiagnoses, and the lack of adult data, is essential for maintaining and improving accuracy. The index will be updated regularly as new data become available.

**Supplementary Information:**

The online version contains supplementary material available at 10.1007/s11892-023-01520-4.

## Introduction

There is an urgent need for improved type 1 diabetes (T1D) epidemiology data across the world, in order to guide and inform resource allocation and health professional training and thereby improve outcomes. Data on T1D incidence, prevalence, or mortality is absent in many countries, and, even when there are data, they are often dated [1•, 2, 3]. Furthermore, although onset of clinical T1D can occur throughout the lifespan, epidemiological data is concentrated on T1D in children and youth and is scarce for adults [2, 4].

This problem of missing data is most pronounced in low- and middle-income countries where access to care is limited, and complications and mortality rates are thought to be high due to marked differences in diabetes diagnosis rates and treatment [2, 5–7].

The T1D Index project of the JDRF, Life for a Child, International Society for Pediatric and Adolescent Diabetes (ISPAD) and the International Diabetes Federation (IDF) was designed to produce just such an understanding by providing estimates of T1D prevalence and mortality in all countries, both now and into the future [8•, 9**•**]. It is an evolving, open-source project aimed at addressing data shortages through an innovative simulation-based approach, which will be updated regularly as new data become available. The initial findings have been published recently [8•] and were used in a 2022 update for the IDF Atlas [10]. These results emphasise the need for timely diagnosis of T1D and improved access to higher levels of care, which could together save millions of lives in coming decades.

## Methodology

At the heart of the T1D Index is a Markov model that estimates the total number of people who have been diagnosed with or died from T1D in any given year based on incidence, mortality, and population size. In simple terms, historical data are used to follow a birth cohort of individuals throughout their simulated lives, with particular proportions of a given cohort developing symptomatic T1D, dying from T1D, or dying from other causes each year, based on the incidence and mortality data available for that year.

Whilst the concept of a Markov model is mathematically sound and frequently used in epidemiologic studies [8•, 11], the outputs of such a model (namely, estimates of prevalence and life-expectancy, and quantities derived from counter-factual scenarios such as “missing prevalence”) can only be as accurate as the model’s inputs allow. In particular, to provide a full set of prevalence estimates in a given year for individuals under the age of 80 years (y), the Markov model requires estimates of population, incidence, and mortality in each country, in each year, and at each age going back at least 80 y prior to the year in question. In addition, to project future prevalence estimates, the model requires projections of population, incidence, and mortality as inputs.

Determining estimates of incidence with this degree of granularity requires access to detailed data on incidence and population denominators in narrow age bands. Some data on diagnosed incidence (under age 20 y) for approximately half of the world’s countries are available from the literature. Data on adult incidence in 32 countries were obtained from Harding and colleagues [4]. However, although T1D incidence has been changing over time [3], data on long-term historical incidence are only available for relatively few countries. This first edition of the T1D Index used ratio-based imputation and stratified regression to extrapolate estimates based on these limited available data.

Producing mortality estimates poses similar challenges in terms of data limitations, requiring estimates of both background and diabetes-related mortality in each country, for each decrement of age and year. Whilst estimates of background population mortality are readily accessible from the UN World Population Prospects [12], it is much less clear how to model mortality arising from T1D, particularly historically. The approach taken in the T1D Index separates T1D-associated mortality into two categories: death soon after onset of symptomatic T1D from non-diagnosis and death in individuals living with a T1D diagnosis. Deaths from non-diagnosis were estimated based on a survey conducted by JDRF and ISPAD in 2020. These were modelled for those younger than 25 y in all countries except for high-income countries (where such deaths were assumed to be zero). Death in individuals living with T1D was estimated as a standardised mortality ratio (SMR) relative to the population mortality. These estimates were derived from a machine-learning method that, for countries and years without published data, predicted SMR based on region, country income class, infant mortality rate, doctors per capita, gross domestic product (GDP) per capita, mortality <5 years, and urbanisation percentage..

Not only is this approach novel but separating the two types of T1D-related deaths allows a quantification of the relative impacts of interventions targeting accurate diagnosis as compared to improving the quality of chronic care. Furthermore, modelling diabetes-related mortality in diagnosed patients based on an SMR allows the index to be integrated with previous work that has estimated SMRs for a given mean HbA1c [2]. The T1D Index can then be used to model the impact of certain T1D interventions such as the provision of insulin and blood glucose test strips on any of the model outputs (e.g., prevalence, life expectancy) by estimating the impact of these interventions on mean HbA1c and thereby on SMR.

## Key Initial Findings

The model estimated that there were 8.4 million prevalent cases of T1D in 2021, with 1.5 million (18%) of these in people <20 y [8••]. The majority of all-age prevalent cases lived in just 10 countries (USA, India, Brazil, China, Germany, UK, Russia, Canada, Saudi Arabia, and Spain).

In 2021, there were an estimated 510,000 incident cases, with 194,000 <20 y [8••].

The results showed that exclusive focus on T1D in the paediatric and young adult population overlooks a significant portion of the T1D global burden. Adult-onset T1D is, in fact, more common than childhood-onset. The median age at diagnosis is 29 y, and the median age of a person living with T1D is 39 y. Although there is an early peak in the age of onset around age 10–14 y, incidence in adults remains substantial with even a potential trend towards an increasing incidence after age 50 y [8••].

**Fig. 1:**
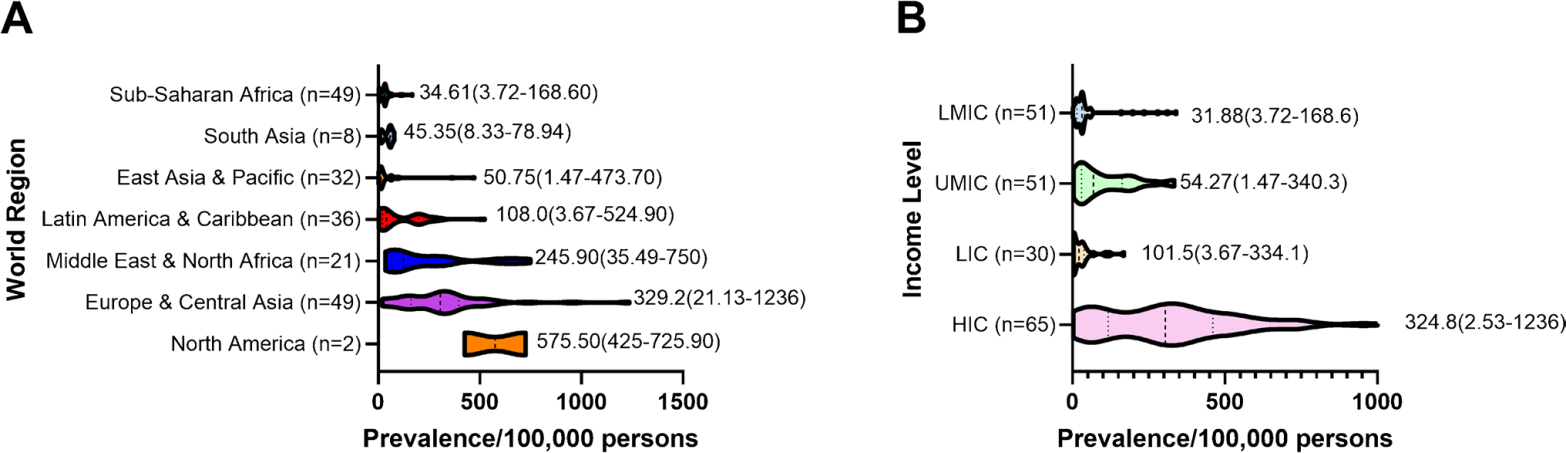
Type 1 diabetes prevalence per 100,000 population (all ages), by **A** world region and **B** income level, 2021. LIC, lower-income countries; LMIC, lower-middle-income countries; UMIC, upper-middle-income countries; HIC, higher-income countries

Prevalence per 100,000 population in 2021 <20 y varied significantly around the world, from 1.5 in Papua New Guinea to 534 in Finland. Table 1 gives 2021 prevalence estimates for all countries for <20 y and all ages, and Fig. 1 shows prevalence by world region and income group. Table 1 in the Supplementary Materials provides prevalence/100,000 for various age groups for all countries.

**Table 1:** Type 1 diabetes prevalence and incidence for all countries, 2021

Country	Prevalence		Type of diabetes	Incidence			
	Prevalence <20 y/100,000	Prevalence all ages/100,000	% of diabetes that is T1D, 20–79 y	T1D Index incidence/100,000 < 15 y	IDF Atlas incidence/100,000 < 15 y	Mid-year of IDF Atlas data	% that IDF Atlas is lower (greater) than T1D Index
Afghanistan	24.8	23.5	0.25	3.5	2.6	2009 (Uzbekistan)	35
Albania	103.4	146.2	1.42	14.7	7.7	2011 (Macedonia)	91
Algeria	252.9	340.3	5.29	40.3	34.8	2015–2017	16
Angola	19.1	33.4	1.3	2.2	1.8	2013 (United Republic of Tanzania)	22
Antigua and Barbuda	89.4	123	1.12	10.9	3.5	1991	211
Argentina	113.6	179.9	3.54	15	6.8	1995	121
Armenia	91.2	116	2.01	13.4	7	2010	91
Aruba	3.6	4.9	0.1	0.3	0.1	1992 (Venezuela)	200
Australia	213.5	473.7	6.89	25.9	24.6	2015	5
Austria	194.6	349.8	5.96	20.9	21.7	2015	(4)
Azerbaijan	67.3	84	1.63	10.1	7.1	2015	42
Bahamas	160.1	212.7	2.53	18.3	10.1	2002	81
Bahrain	73.9	121	1.5	11.7	2.5	1994 (Oman)	368
Bangladesh	9.8	14.6	0.14	1.2	1.3	2016	(4)
Barbados	160.4	210.3	1.26	18.3	5	1987	268
Belarus	128.7	163.3	2.57	18.9	5.6	2000	238
Belgium	171.7	358.7	8.65	19.8	18.1	2011	9
Belize	24.7	36.8	0.34	3.5	3.2	2016 (Mexico)	9
Benin	16	27.6	4.65	1.9	1.7	2016 (Gabon)	12
Bhutan	56.8	58.2	0.68	7.3	4.9	2009 (India)	49
Bolivia	21.9	22.3	0.41	2.9	2.2	2011	32
Bosnia and Herzegovina	132	182.6	1.65	18.4	8.2	2004	124
Botswana	13.9	37.2	1.08	1.6	1.2	2009 (Rwanda)	33
Brazil	184	263.7	2.84	21.9	16.3	2011	34
Brunei Darussalam	14.5	21.3	0.21	1.7	1	2003 (Thailand)	75
Bulgaria	223.3	326.8	3.63	33.6	9.4	1995	257
Burkina Faso	2.8	5	0.45	0.4	0.4	2012 (Mali)	5
Burundi	9.9	16.3	0.59	1.5	1.2	2009 (Rwanda)	25
Cambodia	12.6	12.7	0.22	1.7	1	2003 (Thailand)	75
Cameroon	16.8	28.2	0.84	1.9	1.7	2016 (Gabon)	12
Canada	395	725.9	7.87	42.9	37.9	2016	13
Cape Verde	20.7	49.4	3.2	1.9	1.7	2016 (Gabon)	12
Central African Republic	2.9	4	0.12	0.4	0.4	2012 (Mali)	5
Chad	2.5	3.8	0.15	0.4	0.4	2012 (Mali)	5
Channel Islands	270	537.1	7.65	29.3	28.1	2018 (UK)	4
Chile	125.1	183.5	1.63	16.6	13.9	2016	19
China	19.1	29.8	0.26	2.5	1.9	2012	30
China, Hong Kong SAR	41	63.2	0.61	5.2	4.4	2015	19
China, Macao SAR	40	64.2	0.73	5.2	4.4	2015 (Hong Kong)	19
China, Taiwan Province of China	48.8	90	0.78	5.2	5.2	2012	0
Colombia	22.9	33.9	0.4	2.9	2.2	2008	32
Comoros	24.5	33.6	0.55	3.8	1.4	1992 (Mauritius)	171
Costa Rica	26.8	42.7	0.49	3.4	2.2	2008 (Colombia)	55
Cote d’Ivoire	16.8	27	2.11	1.9	1.7	2016 (Gabon)	12
Croatia	242.4	330.9	5.19	35	17.2	2008	103
Cuba	47.1	80.1	1.01	6.1	2.3	1995	165
Curaçao	3.7	5.1	0.03	0.5	0.1	1992 (Venezuela)	400
Cyprus	145.8	326.4	3.93	14.5	14.4	2005	1
Czech Republic	237.6	394.5	4.51	33.9	21.8	2011	56
Democratic People’s Republic of Korea	36	40.1	0.43	5.1	4.8	2016 (Republic of Korea)	7
Democratic Republic of the Congo	10.5	18.7	0.62	1.5	1.2	2009 (Rwanda)	25
Denmark	248.6	554.5	8.84	28.7	27	2011	6
Djibouti	105.1	197.4	2.93	12.2	11.4	2019 (Eritrea)	7
Dominican Republic	9.6	12.4	0.11	1.2	0.5	1997	140
Ecuador	21.8	32.1	0.82	2.8	2.2	2008(Colombia)	27
Egypt	30.2	35.5	0.22	3.9	3.1	2011	26
El Salvador	24	26.7	0.41	3.5	3.2	2016 (Mexico)	9
Equatorial Guinea	17.7	40.1	1.17	1.9	1.7	2016 (Gabon)	12
Eritrea	100.7	168.6	4.37	12.2	11.4	2019	7
Estonia	323.4	463.6	5.83	42.9	17.1	2003	151
Ethiopia	6.8	15.7	0.75	0.5	0.3	2002	67
Fiji	10.6	12.9	0.08	1.3	0.9	2007	44
Finland	534.2	1235.9	14.68	55.2	52.2	2017	6
France	159.5	299.9	4.03	18.3	18.9	2015	−3
French Polynesia	11.2	15.5	0.07	1.3	0.9	2007 (Fiji)	44
Gabon	17.3	40.3	0.93	1.9	1.7	2016	12
Gambia	2.7	5.2	0.5	0.4	0.4	2012 (Mali)	0
Georgia	112.1	105.8	1.59	16.7	4.6	1999	263
Germany	253.4	503.2	5.7	28.6	24.3	2011	18
Ghana	17.7	32.3	2.3	1.9	1.7	2016 (Gabon)	12
Greece	127.3	257.8	3.06	13.5	15.8	2014	(15)
Grenada	149.5	197	1.68	18.5	5	1987 (Barbados)	272
Guam	1.7	2.5	0.01	0.4	0.1	1998 (Papua New Guinea)	300
Guatemala	24	34.8	0.38	3.5	3.2	2016 (Mexico)	9
Guinea	2.9	5.2	0.47	0.4	0.4	2012 (Mali)	0
Guinea-Bissau	2.8	4.5	0.37	0.4	0.4	2012 (Mali)	0
Guyana	3.4	3.7	0.04	0.4	0.1	1992 (Venezuela)	300
Haiti	7.8	6.9	0.08	1.2	0.5	1997 (Dominican Republic)	140
Honduras	24	29.9	0.75	3.5	3.2	2016 (Mexico)	9
Hungary	235.3	369.5	4.51	31.9	20.1	2011	59
Iceland	169.5	399.2	5.76	20.7	18.2	2009	14
India	56.9	58.8	0.73	7.3	4.9	2009	49
Indonesia	13.8	14.4	0.14	1.7	1	2003 (Thailand)	75
Iraq	60.4	91.3	1.27	8.7	3.2	1994 (Jordan)	172
Ireland	260.2	514.8	15.1	30	27.5	2011	9
Islamic Republic of Iran	70.7	116.8	1.47	9.6	3.7	1994	159
Israel	131.4	260.6	3.33	15.5	14.9	2007	4
Italy	114.4	305.7	3.53	12.8	16.2	2007	(21)
Jamaica	45.7	68.2	0.69	6	2.3	Cuba	161
Japan	25.4	61.8	0.6	3.1	2.2	2008	41
Jordan	29.8	60.8	0.57	3.7	3.2	1994	16
Kazakhstan	17.3	26.1	0.47	2.4	1.9	2012 (China)	25
Kenya	22.1	39.3	1.89	2.2	1.8	2013 (United Republic of Tanzania)	22
Kiribati	9.7	10.1	0.05	1.4	0.9	2007 (Fiji)	56
Kuwait	464.3	750	3.34	71	41.7	2012	70
Kyrgyzstan	17	21.1	0.37	2.4	1.9	2012 (China)	25
Lao People’s Democratic Republic	13	13.3	0.27	1.7	1	2003 (Thailand)	75
Latvia	167.6	251.2	3.32	24.8	7.5	1997	231
Lebanon	30	72.2	1.05	3.6	3.2	1994 (Jordan)	13
Lesotho	20.8	28.5	0.87	2.2	1.8	2013 (United Republic of Tanzania)	22
Liberia	3	5.4	0.43	0.4	0.4	2012 (Mali)	5
Libya	205.1	332.5	4.45	34.3	9	1996	281
Lithuania	211.9	315.9	3.71	31.7	19.9	2011	59
Luxembourg	177.4	373.6	5.94	20.3	18.6	2011	9
Madagascar	19.8	35.2	1.33	2.2	1.8	2013 (United Republic of Tanzania)	22
Malawi	20	30.8	0.81	2.2	1.8	2013 (United Republic of Tanzania)	22
Malaysia	14.2	20.7	0.12	1.7	1	2003 (Thailand)	75
Maldives	60.4	78.9	1.28	9	9.7	2016	(7)
Mali	2.3	3.7	0.33	0.3	0.4	2012	(21)
Malta	227.9	557.7	5.75	22.2	21.9	2008	1
Mauritania	17.8	36.1	2.87	1.9	1.7	2016 (Gabon)	12
Mauritius	33.3	60.9	0.27	4.1	1.4	1992	193
Mayotte	28.3	44.9	1.5	4	1.4	1992 (Mauritius)	186
Mexico	25.7	68.6	0.55	2.8	3.2	2016	(13)
Mongolia	16.5	19.9	0.3	2.4	1.9	2012 (China)	25
Montenegro	240	334.1	3.09	32.8	18.5	2011	77
Morocco	270.9	310.9	3.43	40.8	34.8	1) 2013–2017 2) 2015–2019 (Algeria)	17
Mozambique	19.5	29.8	1.74	2.2	1.8	2013 (United Republic of Tanzania)	22
Myanmar	13.4	12.2	0.18	1.7	1	2003 (Thailand)	75
Namibia	21.8	47.2	1.27	2.2	1.8	2013 (United Republic of Tanzania)	22
Nepal	57.1	57.4	0.92	7.3	4.9	2009 (India)	49
Netherlands	210.4	457.7	7.82	23.8	21.2	2011	12
New Caledonia	11.3	16.1	0.07	1.3	0.9	2007 (Fiji)	44
New Zealand	177.5	362.1	5.51	20.6	19.4	2017	6
Nicaragua	23.5	29	0.37	3.5	3.2	2016 (Mexico)	9
Niger	2.5	4.3	0.17	0.4	0.4	2012 (Mali)	0
Nigeria	14.6	22.6	0.85	1.9	1.7	2016 (Gabon)	12
Norway	317.6	737.5	17.84	36.7	33.6	2011	9
Oman	65.1	113.4	1.11	11.3	2.5	1994	352
Pakistan	7.6	8.3	0.03	1	1	2020	0
Panama	25.9	38.2	0.54	3.3	2.2	2008 (Colombia)	50
Papua New Guinea	1.5	1.5	0.01	0.2	0.1	1998	100
Paraguay	14.6	21.5	0.42	2	0.9	1995	122
Peru	10.4	16.6	0.34	1.4	0.5	1992	180
Philippines	13.7	14.1	0.23	1.7	1	2003 (Thailand)	75
Poland	193.6	304.7	3.62	26	18.8	2012	38
Portugal	183.1	373.9	3.29	18.1	13.2	1996	37
Puerto Rico	422.1	524.9	2.86	43.1	16.8	1995	157
Qatar	280.4	674.5	4.62	31.8	38.1	2019	(16)
Republic of Congo	18.1	31.7	0.83	1.9	1.7	2016 (Gabon)	12
Republic of Korea	41.4	65.7	0.84	5.6	4.8	2016	17
Romania	169	215.2	2.8	23.3	10.1	2013	131
Russian Federation	157.6	220.3	3.52	23.5	12.4	2011	90
Rwanda	12.6	25.6	0.86	1.5	1.2	2009	25
Samoa	10.3	13.2	0.22	1.3	0.9	2007 (Fiji)	44
Sao Tome and Principe	19.4	39.2	1.06	1.9	1.7	2016 (Gabon)	12
Saudi Arabia	413.5	635.6	4.14	67.2	31.4	2006	114
Senegal	17.7	33.9	2.2	1.9	1.7	2016 (Gabon)	12
Serbia	177.8	257.6	2.36	25.7	16.9	2015	52
Seychelles	28.3	58.6	0.74	4	1.4	1992 (Mauritius)	186
Sierra Leone	2.7	3.9	0.29	0.4	0.4	2012 (Mali)	5
Singapore	55.8	100.9	0.74	7	2.4	1993	192
Slovakia	263.5	376.3	4.9	37.5	13.6	2001	176
Slovenia	187.8	302.3	3.85	27.4	16.3	2011	68
Solomon Islands	1.6	1.7	0.01	0.2	0.1	1998 (Papua New Guinea)	100
Somalia	79.2	121.1	3.23	11.7	11.4	2011 (Eritrea)	3
South Africa	23.1	50.1	0.59	2.2	1.8	2013 (United Republic of Tanzania)	22
South Sudan	63.8	68.6	1.61	11.4	10.2	2015 (Sudan)	12
Spain	191.9	436.2	3.36	18.8	18.8	2011	0
Sri Lanka	57.8	63	0.68	7.2	4.9	2009 (India)	47
St Lucia	157.4	195.7	1.8	18.2	5	1987 (Barbados)	266
St Vincent and the Grenadines	158.1	198.9	2.57	18.2	5	1987 (Barbados)	266
State of Palestine	26.9	45.9	0.94	3.5	3.2	1994 (Jordan)	9
Sudan	77.4	107.7	0.87	10.3	10.2	2015	1
Suriname	3.5	4.5	0.04	0.4	0.1	1992 (Venezuela)	300
Swaziland	22.1	33.5	1.22	2.1	1.8	2013 (United Republic of Tanzania)	17
Sweden	427.3	955.2	16.18	50.6	44.1	2009	15
Switzerland	126.4	298.6	5.75	14.4	13.4	2011	7
Syrian Arab Republic	27.3	36.9	0.32	3.5	3.2	1994 (Jordan)	9
Tajikistan	31.6	33.8	0.57	5	2.6	2009 (Uzbekistan)	92
Thailand	13.8	15.6	0.14	1.6	1	2003	65
Timor L’Este	12.9	14	0.22	1.7	1	2003 (Thailand)	75
Togo	2.9	4.9	0.38	0.4	0.4	Mali	5
Tonga	10.9	12.8	0.11	1.2	0.9	2007 (Fiji)	33
Trinidad and Tobago	155.3	203.7	1.52	18.3	5	1987 (Barbados)	268
Tunisia	184.7	234.7	2.42	30.2	7.3	1995	314
Turkey	106	161.2	1.19	13.7	11	2012	25
Turkmenistan	97.2	136.7	2.68	13.4	11	2012 (Turkey)	22
Uganda	19.5	31.7	1.34	2.2	1.8	2013 (United Republic of Tanzania)	22
Ukraine	334.4	279.5	3.86	48.4	7.9	1989	513
United Arab Emirates	73.7	124.5	1.11	11.9	2.5	1994 (Oman)	376
UK	264.1	591.9	8.52	29.5	28.1	2018	5
United Republic of Tanzania	19.6	33.6	0.49	2.1	1.8	2013	17
USA	204.9	425	3.72	24.4	25	2015	(2)
US Virgin Islands	184.6	277.3	1.87	19.4	13.8	2006	41
Uruguay	163.4	292.2	3.05	23.5	8.3	1992	183
Uzbekistan	32.8	36.2	0.61	5	2.6	2009	92
Vanuatu	10.1	11.7	0.11	1.3	0.9	2007 (Fiji)	44
Venezuela	3.4	4.6	0.04	0.4	0.1	1992	300
Viet Nam	13.3	14.5	0.26	1.7	1	2003 (Thailand)	75
Yemen	45.6	50.7	1.39	6.9	2.5	1994 (Oman)	176
Zambia	20.8	35.9	0.63	2.1	1.8	2013 (United Republic of Tanzania)	17
Zimbabwe	21	31.2	2.82	2.2	1.8	2013 (United Republic of Tanzania)	22

Diagnosed incidence <15 y ranged from 0.02/100,000 in parts of Melanesia to >50/100,000 in parts of Europe and the Middle East (see Table 1).

Table 1 in the Supplementary Materials provides prevalence and incidence per 100,000 <15 y, 15–19 y, <20 y, and all ages.

In 2021, there were an estimated 175,000 deaths due to T1D, 35,000 (20%) due to non-diagnosis, and 140,000 (80%) related to excess mortality in diagnosed individuals. However, the relative impact of these two types of mortality is reversed in the younger population. Of the 52,600 deaths occurring in individuals aged <25 y, the majority (67%) of deaths were attributed to non-diagnosis. Most of these occurred in Sub-Saharan Africa (14,500 deaths) and South Asia (8,700) [8••].

Estimated life expectancy of a 10-year old who develops new-onset symptomatic T1D varied from 7 years in a few African countries to up to 70 years in high-income countries [8••].

**Fig. 2:**
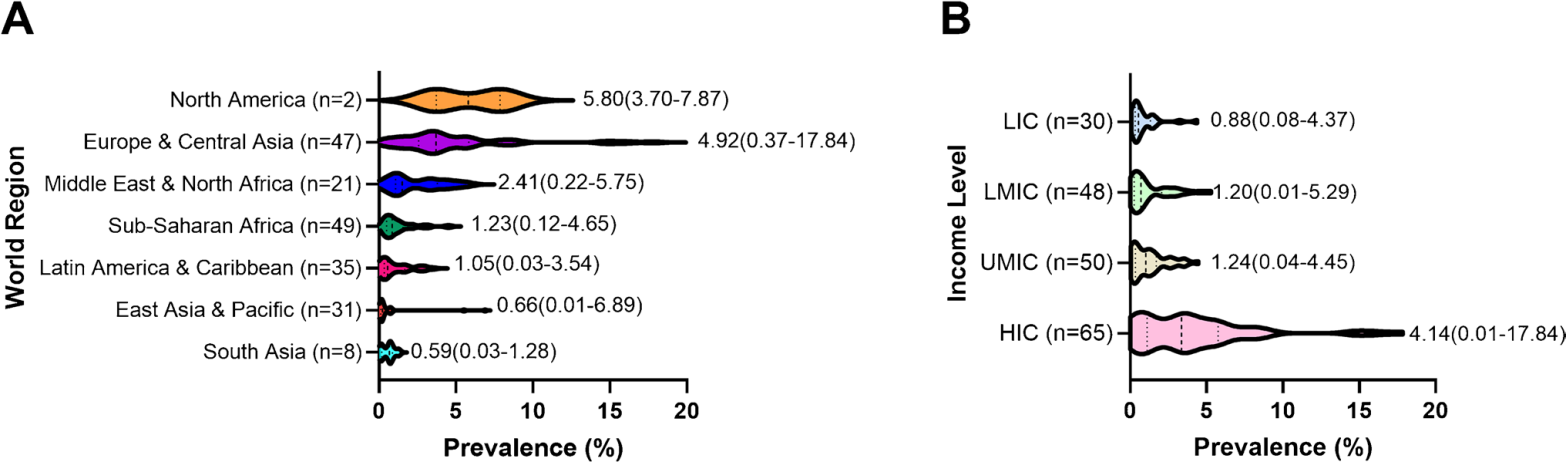
Percentage of all diabetes 20–79 years that is type 1 diabetes, 2021, by **A** world region and **B** income level. LIC, lower-income countries; LMIC, lower-middle-income countries; UMIC, upper-middle-income countries; HIC, higher-income countries

Using the T1D Index prevalence data and the IDF Atlas type 2 diabetes (T2D) prevalence data, T1D as a percentage of all diabetes in the population aged 0–79 years is 1.6% globally (this calculation excludes T2D <20 y as there are no current global estimates of this number). As the incidence of both T1D and T2D vary widely around the world, this percentage varies from 0.01% in Papua New Guinea to 17.8% in Norway. Figure 2 shows the range of T1D as a percentage of all cases of diabetes aged 0–79 y by world region and income group, and Table 1 provides this number for all countries.

## Data Limitations

### Incidence

#### Countries Without Data or With Fluctuating Data

There are few data available on the incidence of T1D; hence, the methods for the T1D Index rely on extrapolation [8••]. For example, only 97 of the 205 countries with more than 50,000 inhabitants have published data for any year at all, and very few countries have any data prior to 1975. Assumptions made about incidence prior to this time naturally affect any estimates made for the population over the age of 45 y. In some countries where data are available, observed incidence rates fluctuate from year to year. In order to use these data to estimate incidence over time in other countries in the same region, these estimates are smoothed. This may partially mask true incidence trends, although it reduces the risk of over-fitting.

#### Incidence Over Time

The work necessitated modelling of changes in incidence over time to populate most data points for most countries. This was done by fitting a line of best-fit to year-on-year annual percentage incidence change data for all years for which two or more countries had data (1985–2015). A global curve was developed, as well as curves for 10 geographic regions where there was sufficient data to model this, which were used in place of the global curves for years that had relevant data.

Table 1 shows the 2021 index incidence per 100,000 population <15 years for all countries, and compares these to the IDF Atlas 10th Edition figures [1•, 13], in which incidence for a particular country was kept stable at the level of the last published data. The median within-country difference between these estimates is an IDF Atlas estimate that was 27.5% lower than the T1D Index estimate. These differences will be greater in low- and middle-income countries if estimated non-diagnosis cases were included as the T1D Index incidence figure would be higher.

These index data show that, whilst estimated incidences have increased in nearly all countries (commensurate with global trends), they have increased three- to five-fold in some countries in Eastern Europe and the Middle East. This is due to the age of some of the studies which date from 1990 to 1999 (including the landmark DIAMOND studies [14]), or even earlier, as well as the rapid year-on-year increases seen in the published data from these regions. Whilst it is reasonable to assume that incidence has risen markedly in these countries with only dated studies countries since the mid-1990s, the 2021 index predictions for countries like Kuwait, Ukraine, and Poland are most likely over-estimated as the projected incidences are now approaching or exceeding incidence in Finland, which has the highest reported incidence. We believe that it is more likely that after incidence has increased steadily for a period, the increase in incidence will then plateau as has been seen in some high-risk European-origin populations [3, 9•]. Further variation would then occur between countries depending on where they are in the timescale depending on the varying temporal impacts of the incompletely understood environmental factors which are causing the increase in incidence. Nonetheless, there is marked inter-country variation in incidence over time even in European-origin populations [3, 9•].

#### Limited Data for Adults, and Misclassification of Diabetes Type

Adult T1D incidence data are only available from 32 countries. The peak age-of-onset appears to be later in sub-Saharan Africa (from a pool of four studies), and so a separate T1D onset pattern was modelled for this region. Moreover, the diagnosis of adult-onset T1D is complicated by the high incidence of T2D in this age group, with a disease progression that is slower and less likely to present with diabetic ketoacidosis, or for insulin treatment to be initiated at onset irrespective of probable type [15]. Misclassification rates of T1D versus T2D are therefore likely to be high [16, 17], and methods of diagnosis vary, depending on the use or availability of biomarkers such as C-peptide and autoantibodies [4].

The potential for misclassification can also occur in childhood and adolescent populations as well, due to the heterogeneity of T1D [18, 19], which appears to be more pronounced in sub-Saharan Africa and South Asia [5, 20].

### Death from Non-diagnosis

It is not possible to obtain data on the death rate from non-diagnosis since unidentified cases by their very nature cannot be reliably enumerated. The rates of death from non-diagnosis used in the index were therefore derived from the JDRF/ISPAD 2020 survey of health professionals, who were asked to estimate, in the region in which they live, the percentage of all children and youth who develop symptomatic T1D who die soon after onset without ever being diagnosed [8••]. They were requested to estimate this rate in three time periods: before 2000, 2000–2010, and after 2010. For example, in Sub-Saharan Africa prior to 2010, the mean survey response was a non-diagnosis rate of 60%.

These numbers may well be underestimates of the number of cases missed. Data from case studies following the initiation of systematic care and increased diabetes awareness can help quantify this issue. For example, in Mali [6], the observed incidence of T1D in the <25-year age group in 2007 was 0.12/100,000, increasing to 0.74/100,000 in 2016, an observed incidence ratio of 6.2. The ratio of observed incidences is equal to the ratio of true incidences multiplied by the ratio of diagnosis rates. Therefore, an increase of this magnitude in the observed incidence can only be due to a substantial increase in true incidence or a substantial increase in diagnosis rate. To explain this increase by true incidence increase alone would require assuming an average annual year-to-year increase in incidence of 22.4% (1.224^6^ = 6.2), far higher than that observed in other countries. A more modest but still rapid increase in true incidence of 7% per year would only give a true incidence ratio of 1.8 (1.07^6^). To explain the observed ratio of 6.2 would require a diagnosis rate in 2016 that was 3.4 times higher than the 2007 rate (6.2/1.8). Even if no cases were missed in 2016 (diagnosis rate of 100%), these figures would imply a diagnosis rate of just 30% (100%/3.4) in 2007. Similar rapid increases in incidence have been observed in Rwanda [21], Gabon [22], and Burkina Faso [23]. See also the Supplementary Material in [8••].

Recently, Ward et al. [24], using a different modelling approach, have also drawn attention to this large number of estimated deaths from non-diagnosis.

### Mortality

Estimates of mortality in individuals living with T1D were based on an SMR relative to background mortality. The SMRs used in the index are based on the outputs of a machine learning model trained on a very sparse dataset consisting of 71 data points representing the SMR for a person with T1D in a particular year, country, and age group. This extrapolation relied on assumptions including that the overall pattern of SMR variation across an individual’s life-span was constant across all countries and years. In addition, one of the inputs on which the model was trained (aside from readily available population statistics such as infant mortality rate, doctors per capita or GDP) was an estimate of the proportion of individuals in a particular region and at a particular time who were receiving a “minimal” versus a more than minimal (“non-minimal”) level of care (minimal care is defined as a simple insulin regimen with minimal or no self-blood glucose monitoring, HbA1c testing, and diabetes education) [2, 7]). These estimates were made based on expert opinion alone (Table 2).

**Table 2:** Estimated number of children and youth <25 years with type 1 diabetes receiving “minimal care” in 2021

Regions/income group	Numbers receiving minimal care	% receiving “minimal care”
East Asia and Pacific	12,096	6
Europe and Central Asia	25,424	5
Latin America and Caribbean	2618	1
Middle East and North Africa	74,640	26
North America	0	0
South Asia	218,238	50
Sub-Saharan Africa	102,071	54
Lower-income countries (LIC)	77,626	65
Lower-middle-income countries (LMIC)	357,461	50
Upper-middle-income countries (UMIC)	0	0
Higher-income countries (HIC)	0	0
Global total	435,087	

### Projections

Projections of future prevalence and associated statistics rely on underlying assumptions about ongoing trends in incidence and mortality which cannot be known with certainty. To account for this, the index modelled two scenarios. In one case (conservative estimate), incidence, mortality, and diagnosis rates were held constant going forward. In the second case (momentum estimate), they continued to change at the average rate of change between 2012 and 2021. Projections suggest that there will be a substantial increase in T1D prevalence between 2020 and 2040, of between 66 and 116% depending on the scenario, due to projected reductions in mortality and, in some regions, increase in incidence. Although the figures are not precise, it is clear that the global burden of T1D will markedly increase in the medium-term future.

## Further Modelling on Health Life Years and Numbers Receiving “Minimal Care”

This work aimed to quantify health-adjusted life years. HALYs, also known as disability-free life-expectancy, is the number of remaining years a person is expected to live without disability. HALYs were quantified by a two-stage procedure. In the first stage, a level of care is assigned to a particular region [2]. A given level of care is assumed to be associated with a particular level of glycaemic control, reflected in the mean achieved HbA1c, and in turn with a particular SMR for diabetes-associated mortality. An iteration of the T1D Index model is run to produce estimates of incidence, prevalence, and mortality for that region. In particular, such an iteration allows estimation of the number of individuals in a particular region who have been living with diabetes for any duration of time.

**Fig. 3:**
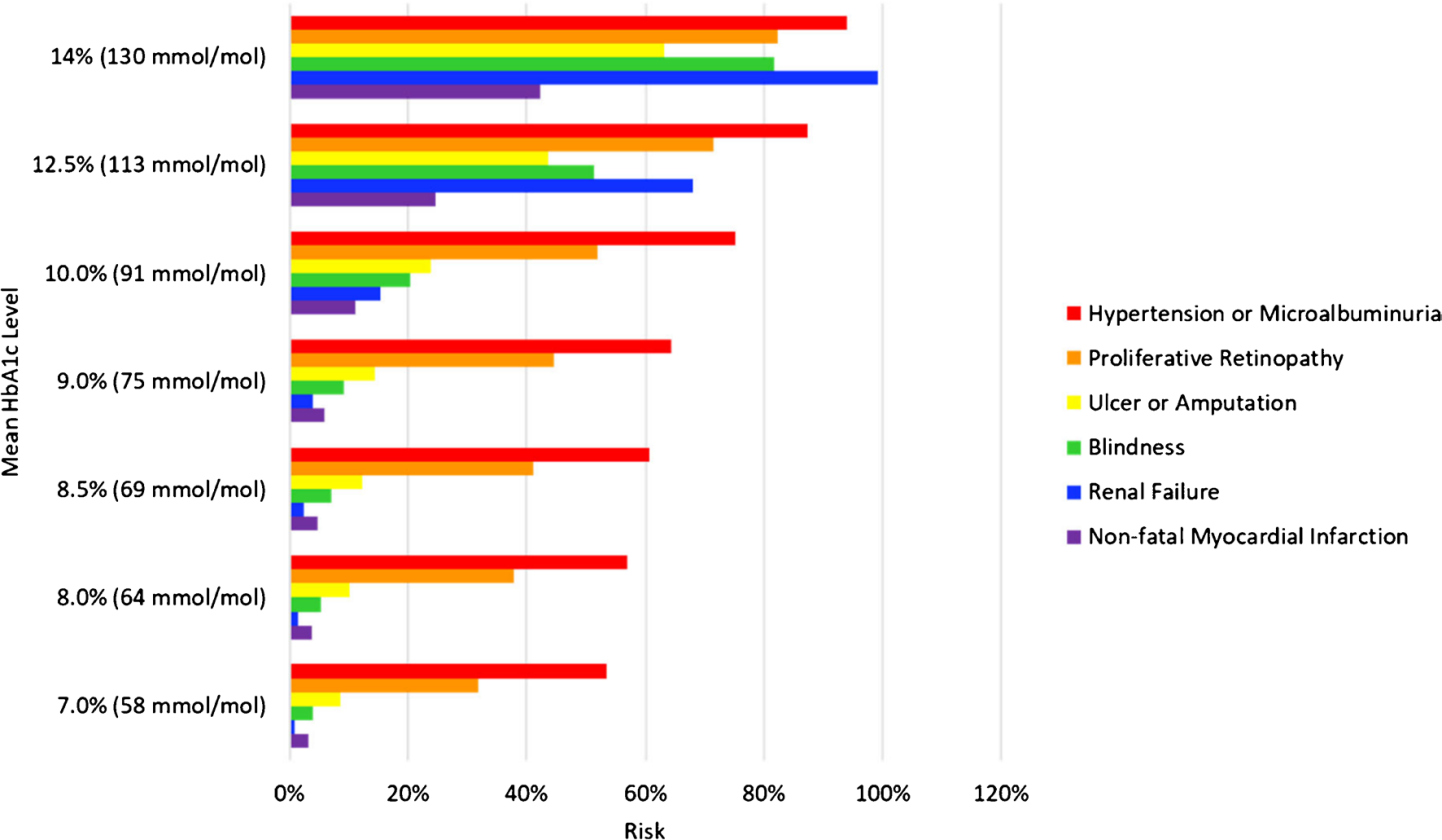
Modelled frequency of selected complications at 30 years after diagnosis of type 1 diabetes. Data from Gregory et al. (2020) [24]

In the second stage, the proportions of individuals living with specific complications of diabetes are determined using methodology from the childhood-onset cohort of the University of Pittsburgh Epidemiology of Diabetes Complications (EDC) study [25•], followed out to 30 years after diagnosis. This methodology provides estimates of the prevalence of several complications at each duration of diabetes, as a function of mean HbA1c. The modelled complications are distal symmetric polyneuropathy, ulcer or amputation, hypertension or microalbuminuria, overt nephropathy, proliferative retinopathy, blindness, non-fatal myocardial infarction, and non-fatal cerebrovascular disease. Each complication type is modelled as a separate occurrence, conditionally independent of one another at a given level of glycaemic control (mean Hba1c). The exception to this principle is an adjustment made to disallow the de novo occurrence of proliferative retinopathy in an already blind individual, and likewise of hypertension/microalbuminuria in an individual with overt nephropathy (such situations were termed ‘impermissible journeys’). Figure 3 is an example of the Gregory et al. [25•] study results, showing the modelled frequency of selected complications at 30 years after diagnosis. Finally, these proportions are combined with population data and standard disability adjusted life years (DALY) costs for each complication to produce an overall estimate of DALYs, reframed as HALYs lost.

**Fig. 4:**
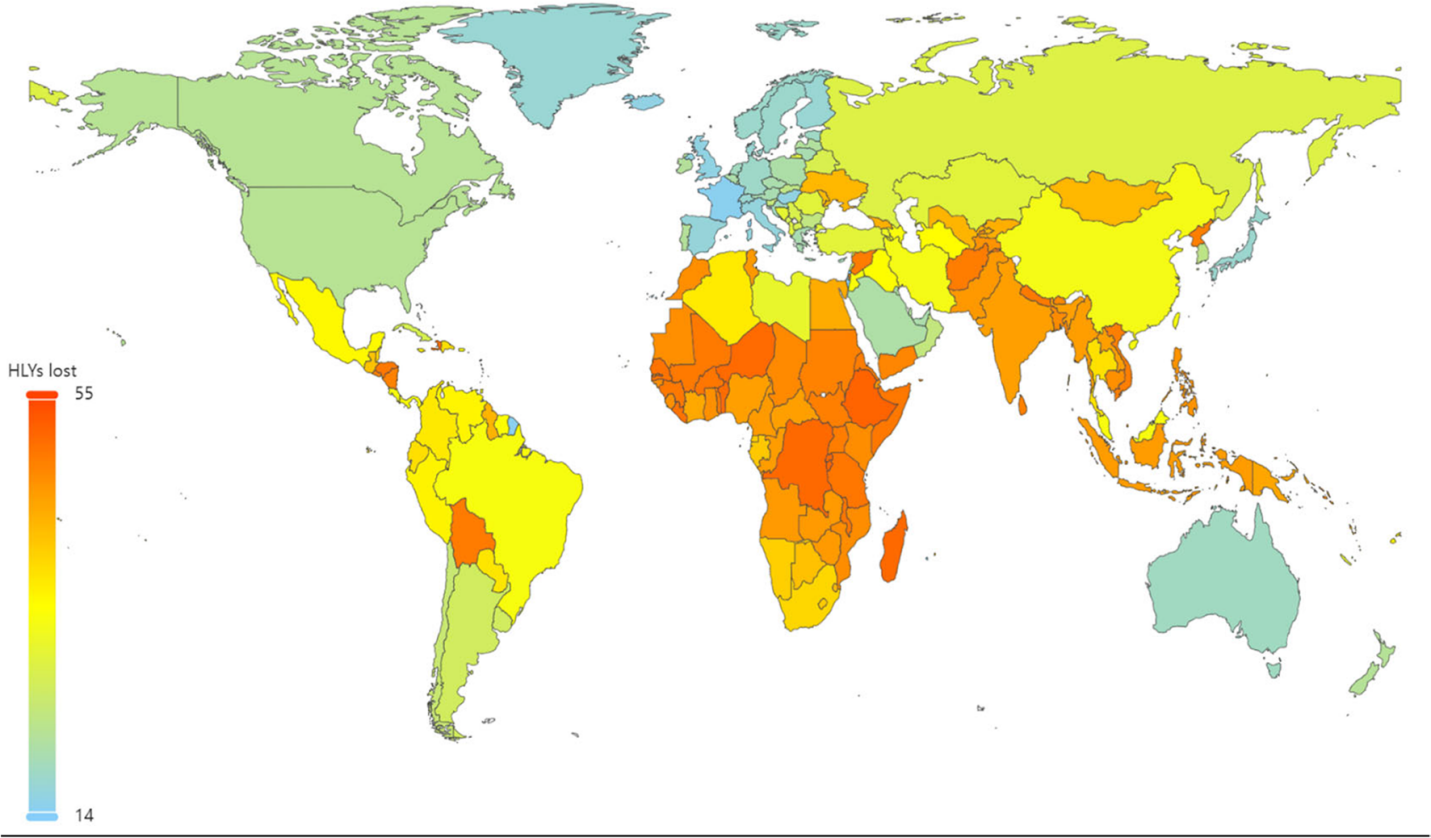
Projected healthy life years lost for an individual with type 1 diabetes diagnosed at age 10 years in 2021

**Fig. 5:**
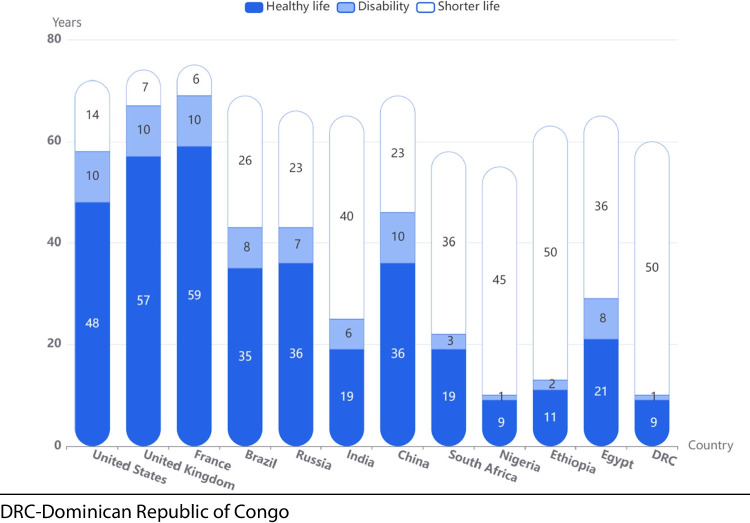
Projected healthy life years lost for an individual with type 1 diabetes diagnosed at age 10 years for 12 representative countries

The analysis showed marked disparities in predicted outcomes for people living with T1D depending on geography. The estimated HLY lost for an individual diagnosed with T1D at age 10 y in 2021 varied from 14 to 55 years (Fig. 4). Figure 5 shows, for 12 representative countries, that the HALY lost in high-income countries are mostly due to disability, whilst in lower-income countries, a shorter lifespan is the major contributor to total HLY lost.

This analysis has a number of limitations. In addition to those noted above regarding the modelling of diabetes-related mortality with SMRs, key assumptions of this modelling are (a) that a particular level of care is able to achieve a given mean HbA1c and (b) that mortality and complication rates can be reliably estimated based on this metric. In practice, the HbA1c achieved by a particular level of care will vary both across individuals receiving that care level, and within patients throughout the duration of their diabetes. For example, the level of glycaemic control tends to vary with age and likely varies across time.

In addition, the EDC study only estimated complication rates up to a diabetes duration of 30 years, and it is not clear what the best assumptions should be for extrapolating ongoing rates of complications beyond 30-year duration. It was also a study of complication rates in a higher-income setting, which may underestimate the complication rates truly experienced by people with diabetes with higher mean HbA1c’s in lower income settings.

## Future Development and Actions

The T1D Index model’s limitations are not static, but rather serve as catalysts for continuous refinement. This improvement is a dual-pronged process involving the integration of new data and the enhancement of the model’s components.

New T1D-specific data will be assimilated into the model at each revision. This includes not just incidence, prevalence, and mortality data but also information on the proportions in each country receiving specific levels of care, and attendant outcomes. These epidemiological and clinical data are becoming more abundant due to the establishment of regional and national registries, the continuous expansion of trans-national initiatives such as the SWEET Database [26], the fostering of epidemiological studies in countries where there are no data or it is quite dated [1•, 27], and data resulting from the operations and attendant research, in partnership with local stakeholders, of programs such as Life for a Child [28], Changing Diabetes in Children [29], and Action4Diabetes [30]. Data collection is becoming easier through digital tools that streamline data collection with in-clinic workflows, using mobile phones and the web. However, there is a recognition [27] that guidelines and tools are needed, such as for standardising the definition of T1D, with resources such as the IDF Guide for Diabetes Epidemiology Studies being potentially helpful in this regard [31].

We will also incorporate changes in underlying country characteristics such as infant mortality, doctor-population ratio, urbanisation rates, and economic changes.

This generation of new data should progressively enhance the accuracy of the index’s predictions. Ideally, as data quality improves and ascertainment becomes complete, reliance on predictions will diminish.

In the interim and beyond integrating new data, we are also committed to refining the model's components. Our strategy involves implementing our own improvements and modifications, which will be open for public comment before integration.

Key areas of methodological improvement we are exploring for future versions of the T1D Index include the following:

Prevalence: Integrating actual prevalence data (e.g., from registries) into the model. Discrepancies between observed prevalence and the model’s predictions will inform adjustments to our overall estimates as well as the inputs and algorithms which shaped them. A “tuning” algorithm of this kind will also mean that including prevalence data from one country will help to refine estimates for similar countries.

Incidence: Through increased access to registry data, as well as new studies and refinements in modelling, we aim to improve our capacity for more accurately projecting out-of-sample data, particularly in settings where a lower incidence (from an older study) could still imply a significantly higher future incidence. We also aim to examine regional differences within large countries such as India.

Diagnosis Rates: Our estimates of non-diagnosis mortality rates described above suggest that the model may currently be under-estimating these rates. We are interested in exploring other estimation methods as well as encouraging and supporting new interventions aimed at improving diagnosis, such as those described in Mali [6].

Mortality and Complication Rates: Recent models predict these rates using published mortality rates and either HbA1C [25•] and/or country characteristics [8••]. Future model revisions will integrate these approaches and include data from registries and administrative data under various monitoring and treatment regimens. This will allow us to improve our estimates of mortality and complication rates, and also improve projections, by leveraging data on the adoption rates of different types and models of care in each country.

We welcome feedback and suggestions for improvement, which can be submitted directly to the authors or to the Index staff at hello@t1dindex.org.

## Conclusions

The first version of the T1D Index has produced robust estimates of T1D incidence, prevalence, and mortality for all countries. The various limitations of the model will be reduced with progressive new versions as more data become available and the modelling is refined.

The results demonstrate great disparity in outcomes around the world. Initiatives are needed to reduce death from non-diagnosis, and improve the level of care, especially in low- and middle-income countries.

### Supplementary Information

ESM 1 (DOCX 52 kb)
