# Correction to: The T1D Index: Implications of Initial Results, Data Limitations, and Future Development

**DOI:** 10.1007/s11892-023-01524-0

**Published:** 2023-09-14

**Authors:** Graham D. Ogle, Gabriel A. Gregory, Fei Wang, Thomas I. G. Robinson, Jayanthi Maniam, Dianna J. Magliano, Trevor John Orchard

**Affiliations:** 1 Life for a Child Program, Diabetes NSW, 26 Arundel St., Glebe, Sydney, New South Wales 2037 Australia; 2 Sydney Medical School, University of Sydney, City Rd, Camperdown, Sydney, New South Wales 2066 Australia; 3 JDRF Australia, 4/80‑84 Chandos St., St Leonards, Sydney, New South Wales 2065 Australia; 4 St Vincent’s Hospital Sydney, 390 Victoria Street, Darlinghurst, Sydney, New South Wales 2010 Australia; 5 School of Medical Sciences, UNSW Sydney, Kensington, Sydney, New South Wales 2052 Australia; 6 Baker Heart and Diabetes Institute, 75 Commercial Rd, Melbourne, Victoria 3004 Australia; 7 School of Public Health and Preventive Medicine, Monash University, 553 St Kilda Rd, Melbourne, Victoria 3004 Australia; 8 Department of Epidemiology, University of Pittsburgh, School of Public Health, Pittsburgh, PA USA

**Correction to: Current Diabetes Reports**

10.1007/s11892-023-01520-4

The original version of this article contained a mistake in the name of the 6th author. It should be Dianna J Magliano rather than Diana J Magliano.

The original article has been corrected.

