# Supplementary material for: The T1D Index: Implications of Initial Results, Data Limitations, and Future Development
Source: Curr Diab Rep. 2023 Aug 23;23(10):277–91. doi: 10.1007/s11892-023-01520-4 (PMC10520097; doi:10.1007/s11892-023-01520-4)
Supplement: Supplementary file 1 — (DOCX 52 kb) [file 11892_2023_1520_MOESM1_ESM.docx]

Supplementary Materials

**Table 1** prevalence and incidence per 100,000 <15 y, 15-19 y, <20 y, and all ages

| **Country** | **Prevalence/100,000** | | | | | **Incidence/100,000** | | | | |
| --- | --- | --- | --- | --- | --- | --- | --- | --- | --- | --- |
|  | **<15y** | **15-19y** | **<20y** | **>20y** | **All ages** | **<15y** | **15-19y** | **<20y** | **>20y** | **All ages** |
| Afghanistan | 20.9 | 38.5 | 24.8 | 22.0 | 5.9 | 3.5 | 3.0 | 3.4 | 2.2 | 6.3 |
| Albania | 79.4 | 165.4 | 103.4 | 159.4 | 1.7 | 14.7 | 12.3 | 14.0 | 9.1 | 1.9 |
| Algeria | 209.9 | 449.3 | 252.9 | 393.2 | 13.5 | 40.3 | 34.6 | 39.3 | 25.5 | 15.0 |
| Angola | 12.3 | 48.1 | 19.1 | 52.3 | 6.7 | 2.2 | 12.8 | 4.2 | 5.6 | 7.4 |
| Antigua and Barbuda | 71.1 | 145.8 | 89.4 | 136.7 | 12.2 | 10.9 | 9.3 | 10.5 | 6.8 | 13.7 |
| Argentina | 84.0 | 205.8 | 113.6 | 211.1 | 16.0 | 15.0 | 12.7 | 14.4 | 9.3 | 18.1 |
| Armenia | 77.0 | 141.7 | 91.2 | 125.0 | 3.7 | 13.4 | 11.4 | 13.0 | 8.3 | 4.3 |
| Aruba | 2.9 | 5.7 | 3.6 | 5.2 | 4.9 | 0.3 | 0.6 | 0.4 | 0.0 | 4.3 |
| Australia | 151.2 | 414.1 | 213.5 | 561.9 | 13.1 | 25.9 | 22.0 | 25.0 | 15.8 | 15.1 |
| Austria | 148.9 | 327.9 | 194.6 | 387.4 | 4.4 | 20.9 | 17.6 | 20.0 | 12.6 | 4.1 |
| Azerbaijan | 57.2 | 105.2 | 67.3 | 91.0 | 4.7 | 10.1 | 8.5 | 9.7 | 6.3 | 5.1 |
| Bahamas | 125.6 | 249.9 | 160.1 | 234.6 | 3.8 | 18.3 | 15.7 | 17.6 | 11.6 | 4.3 |
| Bahrain | 53.6 | 139.0 | 73.9 | 135.6 | 2.2 | 11.7 | 10.2 | 11.4 | 7.5 | 2.8 |
| Bangladesh | 6.2 | 19.9 | 9.8 | 17.3 | 8.6 | 1.2 | 1.0 | 1.2 | 0.8 | 9.7 |
| Barbados | 125.7 | 248.2 | 160.4 | 225.3 | 24.1 | 18.3 | 15.4 | 17.5 | 11.2 | 29.0 |
| Belarus | 108.8 | 200.1 | 128.7 | 173.2 | 5.4 | 18.9 | 16.1 | 18.3 | 11.7 | 4.6 |
| Belgium | 126.7 | 308.6 | 171.7 | 413.0 | 6.6 | 19.8 | 16.8 | 19.1 | 11.9 | 7.6 |
| Belize | 15.7 | 51.0 | 24.7 | 44.5 | 9.0 | 3.5 | 3.0 | 3.4 | 2.2 | 10.6 |
| Benin | 9.9 | 39.9 | 16.0 | 40.4 | 7.8 | 1.9 | 11.2 | 3.8 | 4.8 | 9.0 |
| Bhutan | 43.4 | 94.2 | 56.8 | 58.8 | 0.0 | 7.3 | 6.2 | 7.0 | 4.6 | 0.1 |
| Bolivia | 17.8 | 34.5 | 21.9 | 22.5 | 15.7 | 2.9 | 2.4 | 2.8 | 1.8 | 18.0 |
| Bosnia and Herzegovina | 107.3 | 202.7 | 132.0 | 194.6 | 12.5 | 18.4 | 15.4 | 17.7 | 11.3 | 14.0 |
| Botswana | 6.1 | 40.6 | 13.9 | 54.6 | 6.0 | 1.6 | 8.4 | 3.1 | 3.6 | 6.9 |
| Brazil | 145.9 | 288.4 | 184.0 | 294.3 | 11.3 | 21.9 | 18.6 | 21.0 | 13.7 | 13.0 |
| Brunei Darussalam | 11.5 | 23.1 | 14.5 | 24.2 | 7.1 | 1.7 | 1.4 | 1.6 | 1.1 | 8.0 |
| Bulgaria | 173.3 | 378.2 | 223.3 | 351.5 | 0.8 | 33.6 | 28.0 | 32.2 | 20.3 | 0.9 |
| Burkina Faso | 1.2 | 9.0 | 2.8 | 7.6 | 11.0 | 0.4 | 1.9 | 0.7 | 0.9 | 12.4 |
| Burundi | 4.8 | 32.1 | 9.9 | 24.2 | 11.1 | 1.5 | 8.4 | 2.8 | 3.8 | 12.5 |
| Cambodia | 10.5 | 19.9 | 12.6 | 12.8 | 11.8 | 1.7 | 1.4 | 1.6 | 1.1 | 13.4 |
| Cameroon | 10.5 | 41.1 | 16.8 | 40.7 | 2.2 | 1.9 | 11.1 | 3.8 | 4.8 | 2.6 |
| Canada | 302.0 | 672.5 | 395.0 | 814.0 | 4.6 | 42.9 | 36.1 | 41.2 | 26.5 | 4.1 |
| Cape Verde | 12.4 | 47.2 | 20.7 | 65.8 | 4.5 | 1.9 | 11.1 | 4.1 | 4.3 | 5.3 |
| Central African Republic | 1.3 | 8.4 | 2.9 | 5.5 | 1.8 | 0.4 | 1.9 | 0.8 | 1.0 | 2.1 |
| Chad | 1.1 | 8.0 | 2.5 | 5.6 | 10.7 | 0.4 | 1.9 | 0.7 | 1.0 | 11.9 |
| Channel Islands | 187.4 | 503.2 | 270.0 | 604.5 | 3.6 | 29.3 | 24.9 | 28.1 | 17.9 | 3.3 |
| Chile | 92.2 | 222.7 | 125.1 | 203.4 | 13.4 | 16.6 | 13.9 | 15.9 | 10.1 | 15.4 |
| China | 13.9 | 35.1 | 19.1 | 33.1 | 1.1 | 2.5 | 2.2 | 2.4 | 1.6 | 1.2 |
| China, Hong Kong SAR | 33.0 | 70.4 | 41.0 | 67.6 | 19.3 | 5.2 | 4.4 | 5.0 | 3.2 | 21.5 |
| China, Macao SAR | 32.9 | 70.3 | 40.0 | 69.5 | 0.9 | 5.2 | 4.4 | 5.1 | 3.3 | 0.8 |
| China, Taiwan Province of China | 36.6 | 80.6 | 48.8 | 98.7 | 3.7 | 5.2 | 4.4 | 5.0 | 3.2 | 3.2 |
| Colombia | 16.9 | 39.0 | 22.9 | 38.6 | 1.0 | 2.9 | 2.4 | 2.8 | 1.8 | 1.3 |
| Comoros | 17.3 | 51.7 | 24.5 | 42.3 | 4.7 | 3.8 | 5.0 | 4.1 | 4.7 | 4.2 |
| Costa Rica | 20.0 | 46.6 | 26.8 | 48.8 | 25.9 | 3.4 | 2.8 | 3.2 | 2.1 | 29.0 |
| Cote d'Ivoire | 10.4 | 41.1 | 16.8 | 38.2 | 4.3 | 1.9 | 11.2 | 3.9 | 4.9 | 4.2 |
| Croatia | 194.0 | 388.7 | 242.4 | 351.9 | 0.9 | 35.0 | 29.5 | 33.7 | 21.2 | 0.8 |
| Cuba | 33.7 | 85.1 | 47.1 | 89.0 | 0.9 | 6.1 | 5.1 | 5.8 | 3.7 | 0.8 |
| Curaçao | 2.9 | 5.7 | 3.7 | 5.5 | 17.8 | 0.5 | 0.5 | 0.5 | 0.3 | 19.9 |
| Cyprus | 108.2 | 247.9 | 145.8 | 378.8 | 9.9 | 14.5 | 12.3 | 13.9 | 9.0 | 11.3 |
| Czech Republic | 184.7 | 415.0 | 237.6 | 435.1 | 1.6 | 33.9 | 28.9 | 32.8 | 21.0 | 1.7 |
| Democratic People's Republic of Korea | 23.7 | 70.2 | 36.0 | 41.7 | 3.1 | 5.1 | 4.4 | 4.9 | 3.2 | 3.4 |
| Democratic Republic of the Congo | 5.0 | 34.1 | 10.5 | 29.2 | 3.2 | 1.5 | 8.4 | 2.8 | 3.9 | 3.5 |
| Denmark | 172.1 | 461.4 | 248.6 | 641.4 | 3.2 | 28.7 | 24.4 | 27.6 | 17.6 | 3.5 |
| Djibouti | 57.4 | 256.4 | 105.1 | 253.4 | 1.8 | 12.2 | 53.0 | 22.0 | 24.0 | 2.0 |
| Dominican Republic | 7.7 | 15.6 | 9.6 | 14.0 | 4.6 | 1.2 | 1.0 | 1.1 | 0.7 | 4.3 |
| Ecuador | 16.4 | 38.9 | 21.8 | 37.8 | 2.0 | 2.8 | 2.4 | 2.7 | 1.8 | 2.4 |
| Egypt | 24.8 | 51.7 | 30.2 | 39.3 | 4.7 | 3.9 | 3.4 | 3.8 | 2.5 | 4.2 |
| El Salvador | 15.8 | 48.5 | 24.0 | 28.2 | 20.1 | 3.5 | 3.0 | 3.4 | 2.2 | 22.3 |
| Equatorial Guinea | 11.1 | 45.0 | 17.7 | 59.0 | 3.6 | 1.9 | 11.2 | 3.7 | 4.7 | 4.1 |
| Eritrea | 59.7 | 253.6 | 100.7 | 241.2 | 0.3 | 12.2 | 52.7 | 20.8 | 24.5 | 0.3 |
| Estonia | 271.1 | 498.3 | 323.4 | 501.3 | 9.0 | 42.9 | 36.7 | 41.5 | 25.9 | 10.1 |
| Ethiopia | 2.0 | 23.6 | 6.8 | 24.8 | 20.1 | 0.5 | 7.8 | 2.1 | 2.8 | 22.3 |
| Federated States of Micronesia | 1.3 | 2.6 | 1.6 | 1.8 | 3.2 | 0.1 | 0.0 | 0.1 | 0.0 | 3.6 |
| Fiji | 8.6 | 17.4 | 10.6 | 14.2 | 3.7 | 1.3 | 1.1 | 1.3 | 0.8 | 3.2 |
| Finland | 387.3 | 958.8 | 534.2 | 1424.0 | 17.5 | 55.2 | 46.2 | 52.9 | 33.0 | 19.7 |
| France | 120.7 | 273.3 | 159.5 | 343.2 | 23.5 | 18.3 | 15.4 | 17.6 | 10.9 | 22.8 |
| French Guiana | 131.7 | 283.4 | 166.6 | 294.0 | 0.7 | 20.8 | 17.6 | 20.0 | 13.0 | 0.8 |
| French Polynesia | 8.9 | 17.9 | 11.2 | 17.3 | 1.7 | 1.3 | 1.2 | 1.3 | 0.8 | 2.1 |
| Gabon | 11.2 | 43.4 | 17.3 | 60.1 | 2.4 | 1.9 | 11.2 | 3.7 | 4.5 | 3.0 |
| Gambia | 1.2 | 9.1 | 2.7 | 8.1 | 2.2 | 0.4 | 1.9 | 0.7 | 0.9 | 2.6 |
| Georgia | 95.6 | 172.5 | 112.1 | 103.6 | 4.6 | 16.7 | 14.2 | 16.2 | 10.3 | 4.1 |
| Germany | 183.2 | 457.6 | 253.4 | 561.6 | 24.1 | 28.6 | 24.4 | 27.5 | 17.2 | 22.1 |
| Ghana | 10.9 | 42.4 | 17.7 | 45.3 | 25.1 | 1.9 | 11.1 | 3.8 | 4.6 | 28.3 |
| Greece | 93.2 | 217.5 | 127.3 | 287.3 | 2.7 | 13.5 | 11.2 | 12.9 | 7.8 | 2.4 |
| Grenada | 122.3 | 246.2 | 149.5 | 217.9 | 0.0 | 18.5 | 15.5 | 17.9 | 11.6 | 0.0 |
| Guadeloupe | 117.3 | 231.1 | 150.3 | 210.6 | 0.8 | 17.2 | 14.6 | 16.5 | 10.4 | 1.0 |
| Guam | 1.4 | 2.8 | 1.7 | 2.9 | 32.8 | 0.4 | 0.0 | 0.3 | 0.0 | 37.0 |
| Guatemala | 15.4 | 50.6 | 24.0 | 43.1 | 10.8 | 3.5 | 3.0 | 3.4 | 2.2 | 12.3 |
| Guinea | 1.2 | 9.0 | 2.9 | 7.8 | 12.7 | 0.4 | 1.9 | 0.7 | 1.0 | 15.6 |
| Guinea-Bissau | 1.2 | 8.8 | 2.8 | 6.4 | 0.8 | 0.4 | 1.9 | 0.7 | 0.9 | 0.9 |
| Guyana | 2.7 | 5.4 | 3.4 | 3.8 | 4.5 | 0.4 | 0.3 | 0.4 | 0.3 | 4.1 |
| Haiti | 6.6 | 11.7 | 7.8 | 6.3 | 0.9 | 1.2 | 1.0 | 1.1 | 0.7 | 0.8 |
| Honduras | 15.6 | 48.7 | 24.0 | 33.9 | 9.7 | 3.5 | 3.0 | 3.4 | 2.2 | 11.1 |
| Hungary | 180.0 | 393.1 | 235.3 | 402.0 | 17.1 | 31.9 | 27.1 | 30.6 | 19.5 | 19.0 |
| Iceland | 116.9 | 327.4 | 169.5 | 477.8 | 4.5 | 20.7 | 17.1 | 19.8 | 12.5 | 4.2 |
| India | 43.9 | 93.9 | 56.9 | 59.7 | 7.7 | 7.3 | 6.2 | 7.0 | 4.6 | 8.7 |
| Indonesia | 11.1 | 21.8 | 13.8 | 14.8 | 11.2 | 1.7 | 1.4 | 1.6 | 1.1 | 13.0 |
| Iraq | 42.0 | 127.6 | 60.4 | 119.2 | 10.0 | 8.7 | 7.6 | 8.5 | 5.7 | 11.5 |
| Ireland | 192.2 | 475.1 | 260.2 | 608.8 | 0.0 | 30.0 | 25.1 | 28.8 | 18.2 | 0.1 |
| Islamic Republic of Iran | 52.4 | 139.7 | 70.7 | 137.9 | 2.2 | 9.6 | 8.4 | 9.3 | 6.2 | 2.7 |
| Israel | 93.8 | 266.1 | 131.4 | 331.7 | 0.9 | 15.5 | 13.4 | 15.0 | 9.7 | 0.8 |
| Italy | 80.3 | 205.4 | 114.4 | 346.5 | 0.9 | 12.8 | 10.6 | 12.2 | 7.4 | 0.8 |
| Jamaica | 32.6 | 84.3 | 45.7 | 78.2 | 0.2 | 6.0 | 5.1 | 5.8 | 3.7 | 0.3 |
| Japan | 18.5 | 44.2 | 25.4 | 69.2 | 0.7 | 3.1 | 2.6 | 3.0 | 1.8 | 0.9 |
| Jordan | 20.0 | 60.9 | 29.8 | 83.5 | 2.2 | 3.7 | 3.1 | 3.5 | 2.3 | 2.6 |
| Kazakhstan | 13.5 | 35.0 | 17.3 | 31.0 | 18.7 | 2.4 | 2.2 | 2.4 | 1.6 | 20.8 |
| Kenya | 13.5 | 51.1 | 22.1 | 56.0 | 12.3 | 2.2 | 12.8 | 4.6 | 5.5 | 14.1 |
| Kiribati | 8.1 | 16.0 | 9.7 | 10.5 | 4.5 | 1.4 | 1.0 | 1.3 | 0.9 | 5.3 |
| Kuwait | 373.8 | 795.2 | 464.3 | 855.4 | 1.0 | 71.0 | 59.3 | 68.5 | 43.6 | 1.2 |
| Kyrgyzstan | 13.0 | 33.8 | 17.0 | 23.9 | 5.5 | 2.4 | 2.2 | 2.4 | 1.6 | 6.8 |
| Lao People's Democratic Republic | 10.7 | 20.7 | 13.0 | 13.5 | 18.0 | 1.7 | 1.4 | 1.6 | 1.1 | 20.8 |
| Latvia | 129.8 | 300.6 | 167.6 | 274.4 | 6.1 | 24.8 | 21.6 | 24.1 | 15.4 | 7.1 |
| Lebanon | 19.4 | 61.5 | 30.0 | 92.6 | 9.7 | 3.6 | 3.1 | 3.5 | 2.3 | 11.6 |
| Lesotho | 12.3 | 48.4 | 20.8 | 34.0 | 7.3 | 2.2 | 12.8 | 4.7 | 5.3 | 8.2 |
| Liberia | 1.3 | 9.4 | 3.0 | 7.9 | 3.7 | 0.4 | 1.9 | 0.7 | 0.9 | 4.3 |
| Libya | 139.2 | 425.0 | 205.1 | 403.0 | 1.8 | 34.3 | 28.7 | 33.0 | 21.3 | 2.0 |
| Lithuania | 164.9 | 378.4 | 211.9 | 342.0 | 2.3 | 31.7 | 26.9 | 30.6 | 19.1 | 2.8 |
| Luxembourg | 124.9 | 327.8 | 177.4 | 425.8 | 1.6 | 20.3 | 17.3 | 19.5 | 12.5 | 1.8 |
| Macedonia | 80.1 | 165.6 | 102.4 | 158.2 | 5.3 | 14.6 | 12.2 | 14.0 | 9.1 | 4.9 |
| Madagascar | 12.3 | 47.7 | 19.8 | 50.9 | 0.8 | 2.2 | 12.8 | 4.4 | 5.5 | 1.0 |
| Malawi | 12.5 | 47.4 | 20.0 | 43.5 | 41.2 | 2.2 | 12.8 | 4.4 | 5.6 | 47.5 |
| Malaysia | 11.2 | 23.1 | 14.2 | 23.6 | 1.6 | 1.7 | 1.4 | 1.6 | 1.1 | 1.9 |
| Maldives | 54.4 | 85.3 | 60.4 | 85.0 | 1.0 | 9.0 | 7.7 | 8.7 | 5.7 | 1.3 |
| Mali | 1.0 | 7.5 | 2.3 | 5.7 | 14.8 | 0.3 | 1.6 | 0.6 | 0.8 | 16.6 |
| Malta | 175.8 | 391.2 | 227.9 | 635.1 | 2.3 | 22.2 | 18.8 | 21.4 | 13.6 | 2.7 |
| Martinique | 117.2 | 231.7 | 150.7 | 216.9 | 5.2 | 17.0 | 14.4 | 16.3 | 10.2 | 4.9 |
| Mauritania | 11.0 | 44.3 | 17.8 | 54.2 | 0.9 | 1.9 | 11.1 | 3.8 | 4.7 | 0.8 |
| Mauritius | 21.2 | 60.5 | 33.3 | 69.5 | 20.2 | 4.1 | 5.0 | 4.4 | 4.2 | 24.0 |
| Mayotte | 19.6 | 58.9 | 28.3 | 61.0 | 18.3 | 4.0 | 4.9 | 4.2 | 4.7 | 20.4 |
| Mexico | 14.2 | 59.8 | 25.7 | 90.9 | 12.4 | 2.8 | 2.4 | 2.7 | 1.8 | 13.8 |
| Moldova | 137.3 | 255.0 | 165.7 | 157.7 | 8.6 | 23.2 | 19.7 | 22.4 | 14.6 | 9.6 |
| Mongolia | 12.8 | 33.2 | 16.5 | 22.1 | 5.3 | 2.4 | 2.1 | 2.4 | 1.5 | 4.8 |
| Montenegro | 195.1 | 370.5 | 240.0 | 364.0 | 5.5 | 32.8 | 28.1 | 31.6 | 20.5 | 4.8 |
| Morocco | 218.9 | 442.3 | 270.9 | 331.9 | 1.1 | 40.8 | 34.6 | 39.4 | 25.6 | 1.2 |
| Mozambique | 12.2 | 47.9 | 19.5 | 42.5 | 5.7 | 2.2 | 12.8 | 4.3 | 5.6 | 6.4 |
| Myanmar | 10.8 | 20.3 | 13.4 | 11.5 | 0.8 | 1.7 | 1.4 | 1.6 | 1.1 | 0.7 |
| Namibia | 13.6 | 53.3 | 21.8 | 69.1 | 13.7 | 2.2 | 12.8 | 4.4 | 5.3 | 15.2 |
| Nepal | 43.8 | 92.5 | 57.1 | 57.6 | 10.1 | 7.3 | 6.2 | 7.0 | 4.6 | 11.4 |
| Netherlands | 146.9 | 378.2 | 210.4 | 525.0 | 4.6 | 23.8 | 20.0 | 22.7 | 14.4 | 4.1 |
| New Caledonia | 9.0 | 17.9 | 11.3 | 18.1 | 4.1 | 1.3 | 1.2 | 1.3 | 0.8 | 4.2 |
| New Zealand | 128.3 | 328.5 | 177.5 | 425.5 | 4.6 | 20.6 | 17.4 | 19.8 | 12.6 | 4.3 |
| Nicaragua | 15.6 | 48.9 | 23.5 | 32.3 | 1.8 | 3.5 | 3.0 | 3.4 | 2.2 | 2.1 |
| Niger | 1.1 | 8.8 | 2.5 | 7.1 | 13.8 | 0.4 | 1.9 | 0.7 | 1.0 | 15.4 |
| Nigeria | 9.3 | 36.3 | 14.6 | 32.0 | 1.5 | 1.9 | 11.1 | 3.7 | 4.8 | 1.8 |
| Norway | 224.6 | 584.7 | 317.6 | 863.2 | 19.4 | 36.7 | 30.8 | 35.2 | 22.3 | 22.0 |
| Oman | 50.1 | 138.7 | 65.1 | 131.7 | 24.3 | 11.3 | 10.2 | 11.1 | 7.6 | 28.7 |
| Pakistan | 6.2 | 12.5 | 7.6 | 8.9 | 5.4 | 1.0 | 0.9 | 1.0 | 0.6 | 4.8 |
| Panama | 19.6 | 46.2 | 25.9 | 44.6 | 1.1 | 3.3 | 2.8 | 3.2 | 2.1 | 1.2 |
| Papua New Guinea | 1.3 | 2.4 | 1.5 | 1.4 | 5.3 | 0.2 | 0.2 | 0.2 | 0.1 | 4.8 |
| Paraguay | 10.7 | 27.0 | 14.6 | 25.6 | 4.5 | 2.0 | 1.7 | 1.9 | 1.2 | 5.4 |
| Peru | 7.6 | 20.0 | 10.4 | 19.6 | 14.3 | 1.4 | 1.2 | 1.4 | 0.9 | 16.1 |
| Philippines | 11.2 | 21.5 | 13.7 | 14.4 | 0.8 | 1.7 | 1.4 | 1.6 | 1.1 | 0.9 |
| Poland | 149.1 | 339.0 | 193.6 | 332.5 | 12.4 | 26.0 | 22.1 | 25.1 | 15.9 | 14.3 |
| Portugal | 135.7 | 302.1 | 183.1 | 415.6 | 2.2 | 18.1 | 15.3 | 17.3 | 10.7 | 2.6 |
| Puerto Rico | 347.8 | 582.9 | 422.1 | 553.7 | 0.9 | 43.1 | 36.4 | 41.0 | 26.4 | 0.8 |
| Qatar | 221.6 | 478.1 | 280.4 | 759.4 | 4.7 | 31.8 | 26.9 | 30.7 | 19.9 | 4.1 |
| Republic of Congo | 11.4 | 44.0 | 18.1 | 46.1 | 22.1 | 1.9 | 11.1 | 3.8 | 4.7 | 25.1 |
| Republic of Korea | 27.4 | 78.3 | 41.4 | 70.6 | 7.1 | 5.6 | 4.5 | 5.3 | 3.3 | 8.0 |
| Réunion | 20.2 | 59.5 | 30.5 | 50.1 | 0.6 | 4.0 | 5.0 | 4.3 | 4.2 | 0.8 |
| Romania | 138.0 | 260.1 | 169.0 | 227.4 | 2.0 | 23.3 | 19.7 | 22.4 | 14.2 | 2.4 |
| Russian Federation | 132.3 | 251.2 | 157.6 | 239.5 | 0.1 | 23.5 | 20.0 | 22.7 | 14.5 | 0.2 |
| Rwanda | 5.7 | 38.3 | 12.6 | 38.5 | 1.2 | 1.5 | 8.4 | 3.0 | 3.8 | 1.5 |
| Samoa | 8.4 | 17.7 | 10.3 | 15.8 | 0.9 | 1.3 | 1.1 | 1.3 | 0.9 | 1.0 |
| Sao Tome and Principe | 12.0 | 45.8 | 19.4 | 61.4 | 1.1 | 1.9 | 11.2 | 3.9 | 4.7 | 1.3 |
| Saudi Arabia | 316.6 | 779.6 | 413.5 | 735.5 | 15.2 | 67.2 | 57.8 | 65.2 | 42.5 | 16.9 |
| Senegal | 11.2 | 43.7 | 17.7 | 52.0 | 10.7 | 1.9 | 11.1 | 3.7 | 4.7 | 11.9 |
| Serbia | 133.2 | 294.3 | 177.8 | 279.0 | 25.6 | 25.7 | 21.7 | 24.6 | 15.8 | 28.8 |
| Seychelles | 19.6 | 59.8 | 28.3 | 71.7 | 19.8 | 4.0 | 4.9 | 4.2 | 4.2 | 21.6 |
| Sierra Leone | 1.2 | 8.0 | 2.7 | 5.1 | 4.6 | 0.4 | 1.9 | 0.7 | 0.9 | 4.1 |
| Singapore | 41.5 | 96.8 | 55.8 | 109.9 | 3.2 | 7.0 | 5.9 | 6.7 | 4.3 | 3.6 |
| Slovakia | 207.6 | 443.5 | 263.5 | 405.4 | 4.1 | 37.5 | 31.7 | 36.1 | 23.1 | 4.1 |
| Slovenia | 149.5 | 316.1 | 187.8 | 330.1 | 13.4 | 27.4 | 23.2 | 26.4 | 16.6 | 15.0 |
| Solomon Islands | 1.3 | 2.6 | 1.6 | 1.9 | 13.6 | 0.2 | 0.2 | 0.2 | 0.1 | 15.4 |
| Somalia | 46.0 | 212.8 | 79.2 | 177.9 | 3.7 | 11.7 | 53.0 | 19.9 | 26.6 | 3.3 |
| South Africa | 14.1 | 53.9 | 23.1 | 65.9 | 0.8 | 2.2 | 12.8 | 4.6 | 5.1 | 1.0 |
| South Sudan | 50.9 | 112.8 | 63.8 | 73.6 | 4.6 | 11.4 | 10.6 | 11.3 | 13.1 | 4.1 |
| Spain | 149.1 | 316.1 | 191.9 | 493.8 | 40.3 | 18.8 | 16.0 | 18.1 | 11.3 | 46.8 |
| Sri Lanka | 44.6 | 97.5 | 57.8 | 65.4 | 4.7 | 7.2 | 6.1 | 7.0 | 4.6 | 4.1 |
| St Lucia | 123.1 | 245.9 | 157.4 | 208.1 | 15.0 | 18.2 | 15.3 | 17.4 | 11.4 | 16.7 |
| St Vincent and the Grenadines | 125.3 | 246.8 | 158.1 | 216.1 | 4.1 | 18.2 | 15.3 | 17.4 | 11.4 | 4.1 |
| State of Palestine | 18.3 | 59.5 | 26.9 | 63.5 | 0.9 | 3.5 | 3.0 | 3.4 | 2.3 | 0.8 |
| Sudan | 57.8 | 147.6 | 77.4 | 138.4 | 4.2 | 10.3 | 9.5 | 10.1 | 11.7 | 4.6 |
| Suriname | 2.8 | 5.7 | 3.5 | 5.1 | 22.2 | 0.4 | 0.4 | 0.4 | 0.3 | 24.7 |
| Swaziland | 13.6 | 50.3 | 22.1 | 44.1 | 15.8 | 2.1 | 12.7 | 4.6 | 5.4 | 17.6 |
| Sweden | 311.0 | 802.0 | 427.3 | 1114.1 | 0.1 | 50.6 | 43.2 | 48.8 | 30.8 | 0.2 |
| Switzerland | 91.6 | 233.4 | 126.4 | 341.4 | 26.1 | 14.4 | 12.3 | 13.9 | 8.8 | 22.3 |
| Syrian Arab Republic | 18.3 | 59.3 | 27.3 | 43.1 | 5.0 | 3.5 | 3.1 | 3.4 | 2.3 | 4.8 |
| Tajikistan | 26.9 | 52.2 | 31.6 | 35.6 | 12.7 | 5.0 | 4.2 | 4.8 | 3.2 | 11.8 |
| Thailand | 10.9 | 21.6 | 13.8 | 16.1 | 11.2 | 1.6 | 1.3 | 1.5 | 1.0 | 12.5 |
| Timor L'Este | 10.4 | 21.0 | 12.9 | 14.9 | 4.5 | 1.7 | 1.4 | 1.6 | 1.1 | 5.3 |
| Togo | 1.3 | 8.9 | 2.9 | 7.0 | 11.1 | 0.4 | 1.9 | 0.7 | 0.9 | 12.5 |
| Tonga | 8.8 | 17.5 | 10.9 | 14.3 | 11.1 | 1.2 | 1.2 | 1.2 | 0.8 | 12.9 |
| Trinidad and Tobago | 125.5 | 246.3 | 155.3 | 221.0 | 2.3 | 18.3 | 15.4 | 17.6 | 11.5 | 2.8 |
| Tunisia | 137.5 | 357.0 | 184.7 | 257.1 | 11.7 | 30.2 | 26.3 | 29.3 | 19.4 | 10.9 |
| Turkey | 77.6 | 190.1 | 106.0 | 186.6 | 0.3 | 13.7 | 11.5 | 13.1 | 8.5 | 0.3 |
| Turkmenistan | 73.3 | 188.4 | 97.2 | 161.7 | 5.3 | 13.4 | 11.6 | 13.0 | 8.6 | 4.9 |
| Uganda | 12.3 | 47.9 | 19.5 | 47.9 | 30.4 | 2.2 | 12.8 | 4.3 | 5.7 | 34.4 |
| Ukraine | 286.5 | 497.1 | 334.4 | 265.3 | 8.7 | 48.4 | 41.0 | 46.7 | 29.8 | 9.7 |
| United Arab Emirates | 54.8 | 139.1 | 73.7 | 136.6 | 2.3 | 11.9 | 10.3 | 11.5 | 7.6 | 2.7 |
| United Kingdom | 190.0 | 503.4 | 264.1 | 690.5 | 3.0 | 29.5 | 25.0 | 28.4 | 17.9 | 3.7 |
| United Republic of Tanzania | 12.4 | 48.4 | 19.6 | 50.2 | 1.0 | 2.1 | 12.7 | 4.3 | 5.5 | 1.1 |
| United States | 144.9 | 375.7 | 204.9 | 497.2 | 1.1 | 24.4 | 20.7 | 23.5 | 15.0 | 1.3 |
| United States Virgin Islands | 135.4 | 327.1 | 184.6 | 308.4 | 0.9 | 19.4 | 16.3 | 18.6 | 11.9 | 0.8 |
| Uruguay | 102.6 | 340.0 | 163.4 | 340.5 | 0.8 | 23.5 | 20.2 | 22.6 | 14.4 | 1.0 |
| Uzbekistan | 27.7 | 52.2 | 32.8 | 38.1 | 11.3 | 5.0 | 4.2 | 4.8 | 3.1 | 12.9 |
| Vanuatu | 8.3 | 16.9 | 10.1 | 13.1 | 18.5 | 1.3 | 1.1 | 1.3 | 0.9 | 21.4 |
| Venezuela | 2.8 | 5.4 | 3.4 | 5.2 | 8.3 | 0.4 | 0.3 | 0.4 | 0.3 | 9.8 |
| Viet Nam | 10.9 | 21.8 | 13.3 | 14.9 | 8.4 | 1.7 | 1.4 | 1.6 | 1.1 | 10.0 |
| Western Sahara | 1.3 | 10.0 | 3.3 | 11.0 | 5.5 | 0.4 | 1.8 | 0.8 | 0.8 | 4.8 |
| Yemen | 33.7 | 88.4 | 45.6 | 55.7 | 28.1 | 6.9 | 6.0 | 6.7 | 4.5 | 31.4 |
| Zambia | 12.9 | 50.3 | 20.8 | 54.4 | 7.1 | 2.1 | 12.7 | 4.4 | 5.6 | 7.8 |
| Zimbabwe | 13.4 | 49.3 | 21.0 | 42.5 | 17.8 | 2.2 | 12.8 | 4.4 | 5.5 | 20.2 |
